# Dermal regeneration in full-thickness wounds: Clinical outcomes with novosorb^Ⓡ^ biodegradable temporising matrix

**DOI:** 10.1016/j.jpra.2026.07.017

**Published:** 2026-07-16

**Authors:** Doha Obed, Marcel R. Oelerich, Katharina Flöthmann, Nadjib Dastagir, Frederik Schlottmann, Martynas Tamulevicius, Florian Bucher, Lukas Wellkamp, Peter M. Vogt

**Affiliations:** Department of Plastic, Aesthetic, Hand, and Reconstructive Surgery, Hannover Medical School, Hannover, Germany

**Keywords:** Wounds, Btm, Biodegradable temporising matrix, Novosorb, Dermal regeneration template

## Abstract

Complex full-thickness skin defects remain challenging to reconstruct due to the loss of regenerative dermal structures. Dermal regeneration templates such as the NovoSorb^Ⓡ^ Biodegradable Temporising Matrix (BTM) have been introduced to facilitate dermal regeneration prior to definitive skin grafting. We hereby describe the outcome following its use in the context of wound treatment. A retrospective analysis was conducted including all patients treated with BTM at a single plastic surgery center between March 2020 and February 2026. Demographic, clinical, microbiological, and surgical data were extracted from electronic health records. Outcomes of interest included BTM infection, BTM loss prior to skin grafting and skin graft take. Univariate and multivariable analyses were performed to identify factors associated with BTM loss.

A total of 104 wounds in 86 patients were included. Burns were the most common etiology (40.7%), followed by chronic wounds (19.8%), infections (17.4%), trauma (15.1%), and tumors (9.3%). The mean wound area treated with BTM was 725 cm² (± 937.6). Complete BTM integration occurred in 74.1% of wounds, partial integration in 9.6%, and BTM loss in 16.3%. Infectious complications occurred in 15.4% and skin graft failure following successful BTM integration in 6.9%. Peripheral arterial disease and exposed tendons were significantly more frequent in cases of BTM loss in univariate analysis but were not independent predictors in multivariable analysis. BTM represents a reliable reconstructive option for complex full-thickness wounds across diverse etiologies, demonstrating high integration and graft success rates. Peripheral arterial disease and exposed tendons may increase the risk of BTM loss, although independent predictive value was not confirmed.

## Introduction

Wounds characterized by complete epidermal and dermal loss remain a significant therapeutic challenge in modern wound care.^1^ Such injuries may arise from a wide range of etiologies, including traumatic avulsion or degloving injuries, deep thermal burns, chronic ulcers, severe infections, and necrotizing soft tissue diseases. Owing to the loss of the skin’s regenerative layers, these wounds necessitate specialized reconstructive strategies.^2^^,^^3^

Conventional management of full-thickness wounds centers on prompt and thorough removal of devitalized tissue, followed by optimization of the wound bed and, ultimately, definitive autologous skin coverage. In large defects, split-thickness skin grafts are commonly used to achieve wound coverage. However, these are associated with significant rates of hypertrophic scarring and contracture due to limited dermal replacement, as well as notable donor site morbidity in larger defects.^4^^,^^5^ The pursuit of improved dermal reconstruction strategies has led to the emergence of dermal regeneration templates (DRTs). These constructs, produced from synthetic, xenogeneic, or allogeneic materials, are engineered to provide a provisional matrix that supports neovascularization and subsequent formation of a durable neodermis within the wound bed.^6^^,^^7^ Their use has shown to aid in limiting donor site morbidity and enhancing both functional and aesthetic results in the reconstructive site.^8^^,^^9^

The NovoSorb^Ⓡ^ Biodegradable Temporising Matrix (BTM), developed by PolyNovo Biomaterials Pty Ltd. in Port Melbourne (Australia), is a synthetic dermal substitute intended for use within a staged reconstructive approach.^10^ This DRT is composed of two distinct layers: a porous, biodegradable polyurethane matrix engineered to permit tissue ingrowth, and an overlying non-resorbable membrane that functions as a temporary barrier.^11^

Following meticulous preparation of the wound bed, the matrix is applied to facilitate cellular infiltration, neovascularization, and formation of a stable dermal analog, while concurrently reducing evaporative fluid loss and shielding the wound from external contamination.^12^ Optimal integration depends on thorough removal of nonviable tissue, which may be achieved through a combination of mechanical, or enzymatic debridement techniques, frequently supported by adjunctive modalities such as negative pressure wound therapy (NPWT).^13^ Successful integration of the scaffold is clinically recognized by the presence of capillary perfusion within the matrix structure, which is usually given three to four weeks following initial application.^14^ Once integration is complete, the superficial membrane is removed, and final wound closure is most commonly accomplished using a split-thickness skin graft.

Multiple clinical reports have described favorable results following the application of BTM across a range of clinical scenarios.^15^, ^16^, ^17^ In this study, we present our single-center experience using BTM within a standardized treatment algorithm for multiple indications in a cohort of 86 patients presenting with complex full-thickness skin defects. The primary objective was to assess the clinical performance of this synthetic dermal substitute in the management of challenging full-thickness wounds with particular emphasis on its applicability, integration reliability, resistance to infection, BTM loss and reconstructive outcomes.

## Methods

### Study design and data extraction

A retrospective analysis was performed based on data extracted from a prospectively curated institutional database, encompassing all consecutive cases in which BTM was utilized for reconstruction at our Department of Plastic, Aesthetic, Hand and Reconstructive Surgery. Ethical approval for the study was granted by the Ethics Committee of Hannover Medical School (Ref.: 12,297-BO-K-2026). The study covered the interval spanning March 2020 to February 2026. Each case underwent a thorough evaluation of treatment options attended by at least one consultant surgeon, during which alternative reconstructive strategies were evaluated prior to consensus on the use of BTM. All eligible patients treated during the study period were included, and no exclusion criteria were applied. One wound was excluded from outcome analysis since prior in-hospital mortality did not allow for outcome evaluation. The outcomes focused on the occurrence of BTM infection or loss prior to split-thickness skin grafting, as well as skin graft take. Localized infection of the matrix was identified by clinical signs including peri‑wound erythema and/or the presence of purulent or suppurative exudate beneath the sealing membrane. In addition, systemic indicators of infection, e.g. fever or hemodynamic compromise occurring in conjunction with local signs, were documented.

To support accurate classification of infectious events, microbiological cultures were analyzed from wound swabs obtained prior to BTM placement. Further, anonymized data on demographics, clinical characteristics (comorbidities, wound etiology, exposed structures, application of NPWT, skin graft take rates, size of the total body surface area (TBSA) treated with BTM, time between the application of BTM and skin grafting), as well as in-hospital mortality, admission times and length of stay were extracted from the institution’s internal electronic health record system.

### Surgical management

**Fig. 1:**
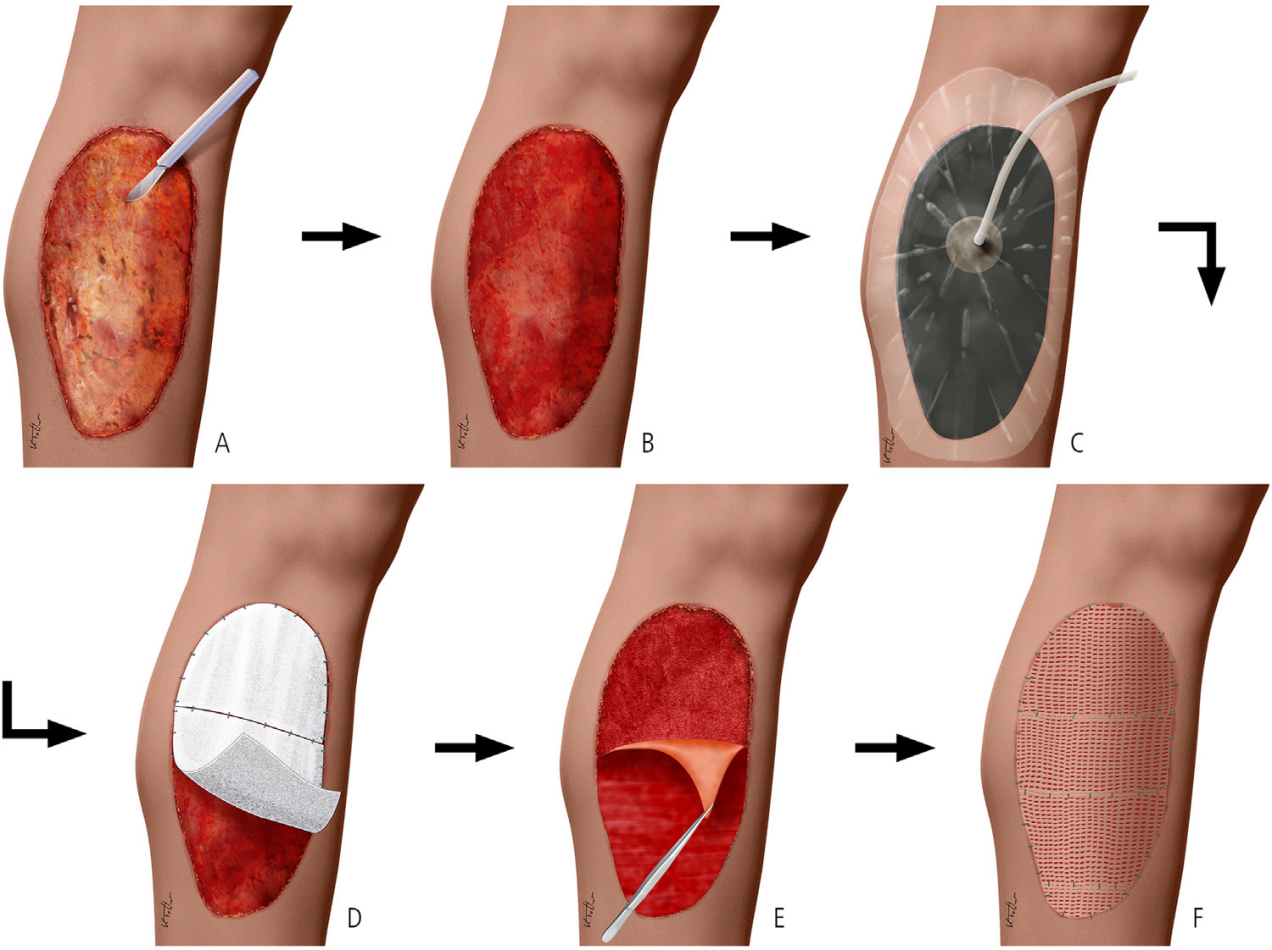
Visualization of the surgical approach to wound bed preparation and BTM application for wound coverage. (A) A surgical debridement of non-viable tissue is conducted (B) until a clean and well vascularized wound bed is achieved. (C) Negative pressure wound therapy may be incorporated to achieve a sufficiently clean wound bed. (D) Application of the BTM onto the wound surface and fixation using skin staples. (E) Delamination (removal of the sealing membrane) following BTM integration. (F) Definitive wound coverage by autologous split-thickness skin grafting.

Management across the study cohort followed a broadly uniform treatment pathway (Fig. 1). Definitive reconstruction was undertaken only once the wound was deemed macroscopically clean and no further surgical debridement was required.

BTM was affixed in all cases using sterile skin staples followed by NPWT or compression bandaging when anatomically appropriate. At each dressing change, the matrix was systematically assessed for evidence of integration, neovascularization, and any procedure-related complications. Routine cleansing of the BTM surface was performed using antiseptic solutions such as povidone-iodine or octenidine and phenoxyethanol. Any hematoma formation was evacuated manually, and meticulous hemostasis was achieved to restore direct apposition between the BTM and the underlying wound surface. In cases where purulent secretion was identified beneath the matrix, the area was thoroughly irrigated, followed by localized antiseptic treatment in conjunction with appropriate systemic antibiotic therapy when deemed necessary.

Progression to the second reconstructive stage was determined once near-complete matrix integration had occurred, defined clinically by a uniform dark pink appearance and consistent capillary refill throughout the matrix. Definitive coverage was achieved exclusively with split-thickness skin grafts applied either fenestrated or meshed and secured with sutures or staples following the removal of the superficial membrane. Post-grafting dressings consisted of a non-adherent fatty gauze combined with povidone-iodine or antiseptic-soaked gauze, dry secondary dressings, and compression bandaging, or alternatively NPWT.

### Statistical analysis

Data were organized using Microsoft Excel (Version 16, Microsoft Corporation, Redmond, WA). Statistical analysis and descriptive statistics were conducted using GraphPad Prism 9 (GraphPad Software Inc., San Diego, CA, USA). Continuous data are presented as mean ± standard deviation (SD), while ordinal data are expressed as counts and percentages. To identify predictors of BTM take, a univariate analysis was conducted. Categorical variables were evaluated using either the chi-square or Fisher’s exact test, while continuous variables were compared using the Student’s *t*-test or Mann–Whitney U test, based on assessment of distribution normality. Variables demonstrating a univariate association at a threshold of p < 0.05 were subsequently entered into a multivariable model. Independent factors associated with BTM loss were then determined through multiple logistic regression analysis. Statistical significance was defined using a threshold of p < 0.05.

## Results

A total of 104 wounds in 86 patients were included in the analysis. Detailed demographic and clinical characteristics are presented in Table 1. The cohort consisted primarily of male patients (66.3%) and the mean age was 51 years (± 18.7). Frequently observed comorbidities included arterial hypertension (33.7%), renal insufficiency (31.4%), diabetes mellitus (19.8%), and peripheral arterial disease (10.5%). Among all individuals, 18.4% (n = 16) were active smokers.

**Table 1:** Clinical, demographic and wound characteristics in all 86 patients.

Variable	Total (*n* = 86)
Age (years), (mean ± SD)	50.7 ± 18.7
Male Gender, n (%)	57 (66.3)
Comorbidities, n (%)	
Arterial hypertension Renal insufficiency (GFR < 90 ml/min) Diabetes mellitus Active smokers Peripheral Arterial Disease	29 (33.7)
	27 (31.4)
	17 (19.8)
	16 (18.4)
	9 (10.5)
Wound Etiology, n (%)	
Burns Chronic wounds Infection Trauma Tumour	35 (40.7)
	17 (19.8)
	15 (17.4)
	13 (15.1)
	8 (9.3)
Exposed structures, n (%)	
Subcutaneous/muscle tissue Tendons Bones	47 (54.7)
	29 (33.7)
	15 (17.4)
Wound Location, n (%)	
Hands	21 (24.4)
Upper limbs	29 (33.7)
Feet	7 (8.1)
Lower limbs	40 (46.5)
Anterior trunk	18 (21.0)
Posterior trunk	10 (11.7)
Head/Neck	13 (15.1)
Burn Etiology, n (%)	
Flame/Contact Scalding Explosion/Deflagration Electricity	18 (51.4)
	2 (5.7)
	9 (25.7)
	2 (5.7)
ABSI (burn patients), (mean ± SD)	8.1 ± 2.8
Total TBSA (%) (burn patients), (mean ± SD)	31.2 ± 22.3

GFR: glomerular filtration rate; TBSA: total body surface area;.

ABSI: abbreviated burn severity index; SD: standard deviation;.

Treatment and outcome characteristics in all 86 patients are presented in Table 2. Among all patients evaluated, 40 (46.5%) received BTM applied as primary wound coverage. Given wounds were older than two weeks in 47.7% of all patients at the time of BTM application. On average, the wound area covered with BTM was 725 cm^2^ (± 937.6). NPWT was applied in 60.5% of all cases following BTM placement. All underlying etiologies were included in the analysis. Burns represented the largest proportion of patients (40.7%), followed by chronic nonhealing wounds (19.8%), infections (17.4%), trauma (15.1%), and tumors (9.3%). The most common burn etiology was flame and contact burn (51.4%), followed by explosion and deflagration (25.7%), scalding (5.7%) and electrical burns (5.7%). In burn patients, the average Abbreviated Burn Severity Index (ABSI) was 8.1 (± 2.8). and the TBSA affected was 31.2% (± 22.3). In total, an in-hospital mortality rate of 4.7% with 4 deaths among the cohort was noted.

**Table 2:** Treatment and outcome characteristics in all 86 patients.

Variable	Total (n = 86)
TBSA covered with BTM in cm2, (mean ± SD)	725.4 ± 937.6
BTM applied as primary wound coverage, n (%)	40 (46.5)
Wound > 2 weeks old at BTM application, n (%)	41 (47.7)
NWPT application, n (%)	52 (60.5)
Positive wound swab prior to BTM application, n (%)	61 (70.9)
In-hospital mortality rate, n (%)	4 (4.7)
LOS from BTM application (days), (mean ± SD)	49 ± 53.7

TBSA: total body surface area; BTM: Biodegradable Temporising Matrix;.

SD: standard deviation; NWPT: negative pressure wound therapy; LOS: length of stay;.

**Fig. 2:**
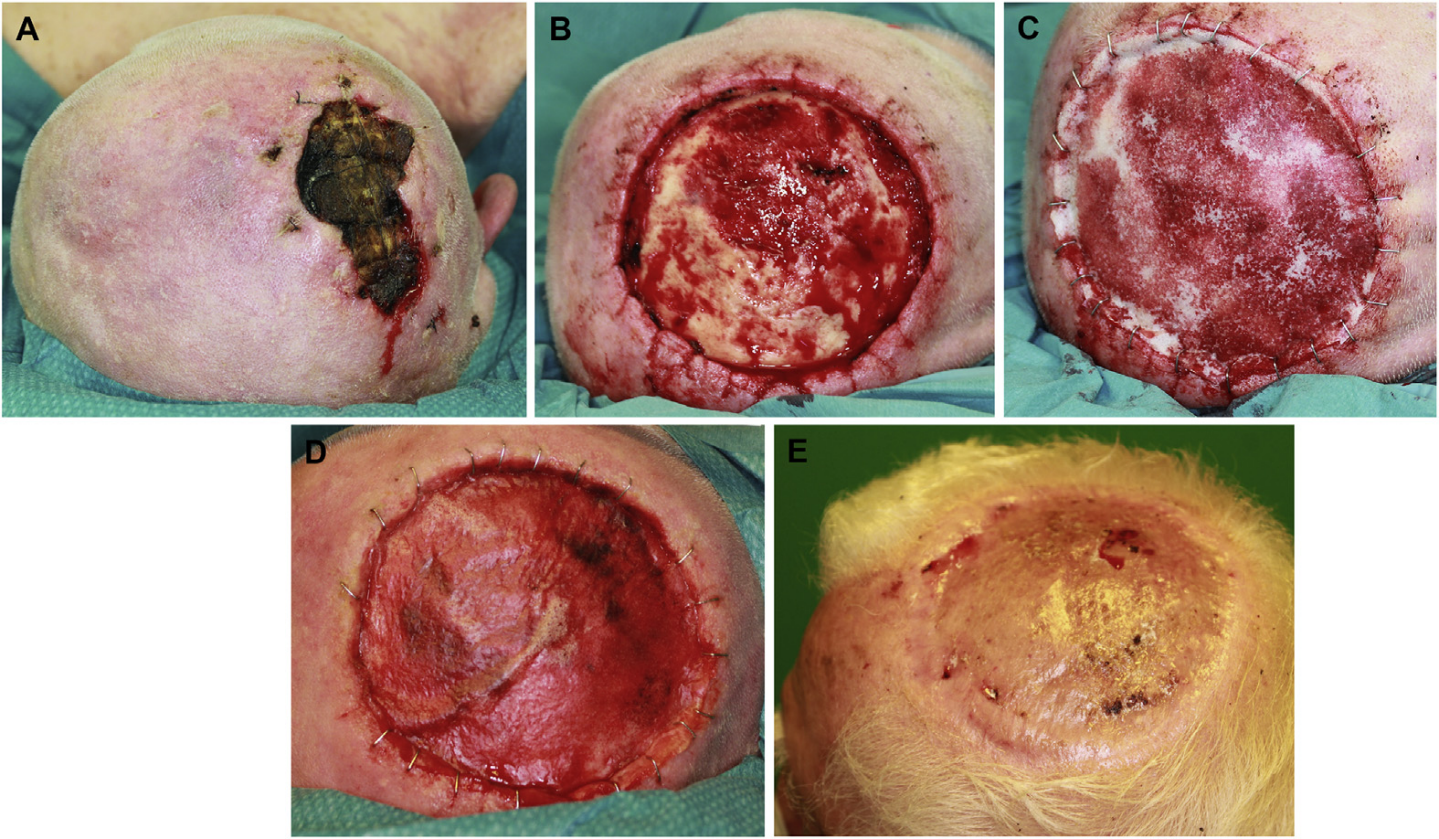
An 82-year-old male following incomplete resection of angiosarcoma in the parietal region. (A) Initial appearance; (B) Appearance following R0-resection with bone abrasion (C) and BTM application. (D) Appearance of vascularized BTM 2 weeks after application; (D) (E) 2-month follow-up after autologous split-thickness skin grafting.

**Fig. 3:**
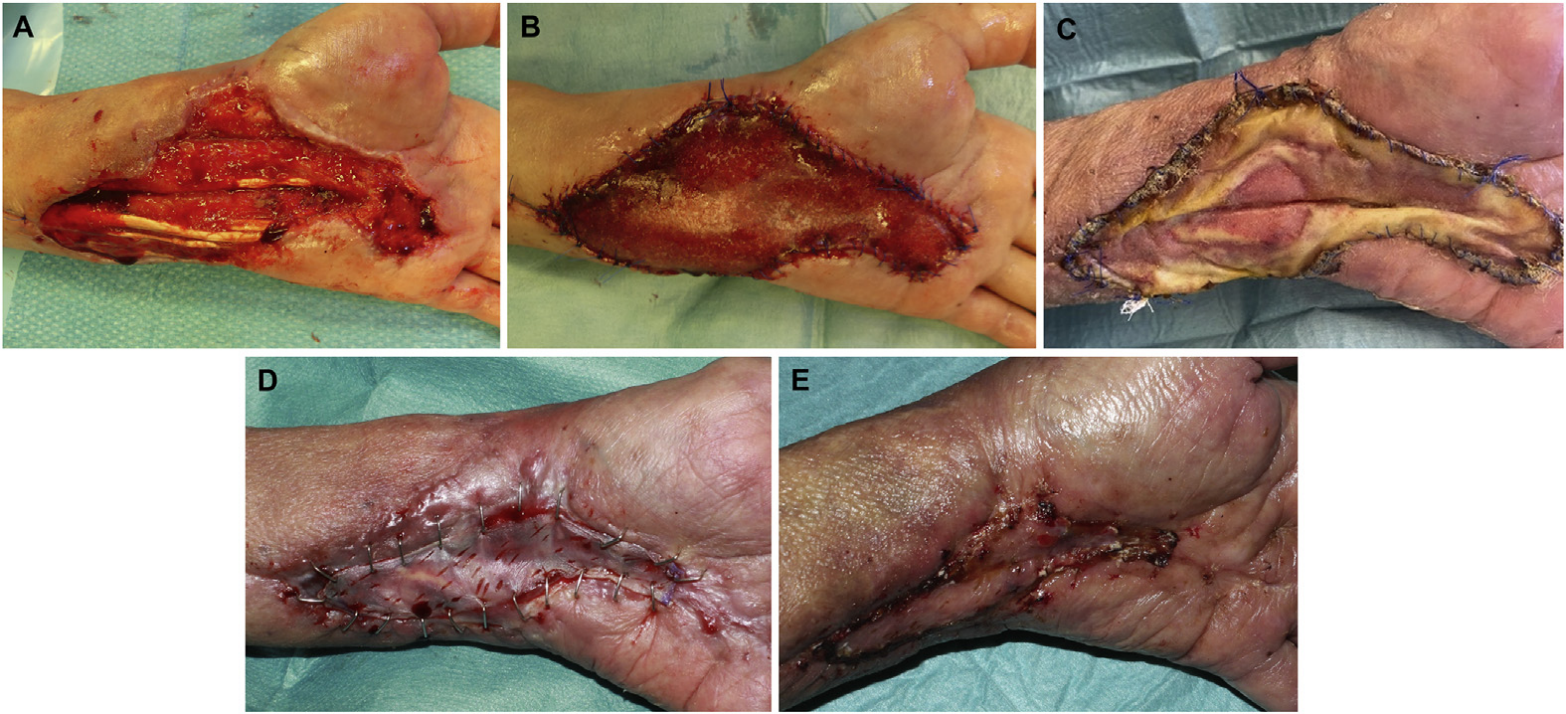
A 49-year-old female with a wound secondary to infection in the palmar hand and distal forearm. (A) Appearance following serial debridement and NPWT; (B) Appearance following BTM application; (C) Appearance of vascularized BTM 4 weeks after application; (D) Autologous split-thickness skin grafting; (C) 2-month follow-up.

Figs. 2 and 3 depict three cases of various indications and their clinical course.

At baseline evaluation, the extent of tissue exposure including subcutaneous or muscle tissue was noted in 54.7% (n = 47) of all patients, with exposed tendons in 33.7% (n = 29) and exposed bone in 17.4% (n = 15) of all patients. The lower limbs and upper limbs were most frequently addressed with BTM (46.5% and 33.7%, respectively).

**Fig. 4:**
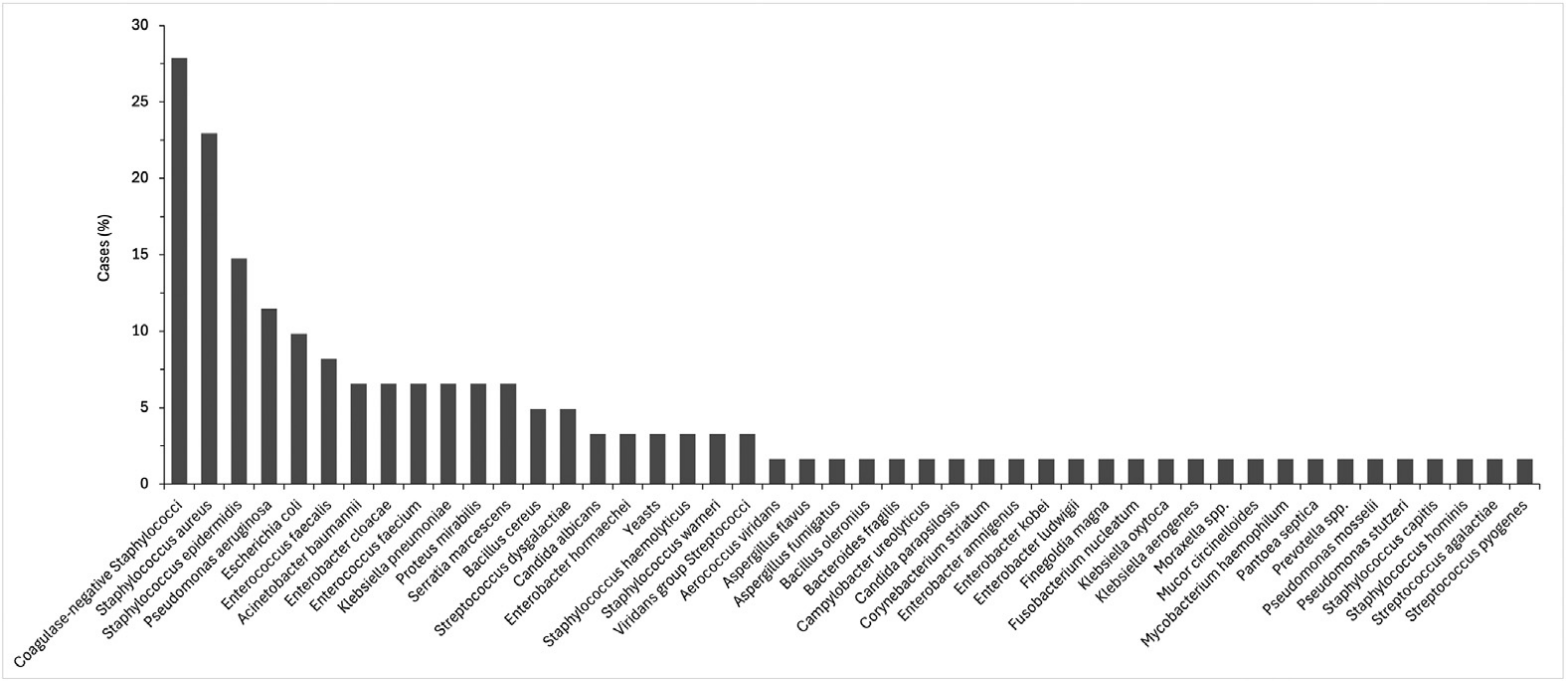
Microbiological colonization distribution from positive wound swab cultures taken prior to BTM application. The figure displays the relative frequency of bacterial and fungal species isolated from contaminated wounds before BTM placement. Despite a high prevalence of preoperative colonization, BTM demonstrated acceptable infection and integration outcomes.

**Fig. 5:**
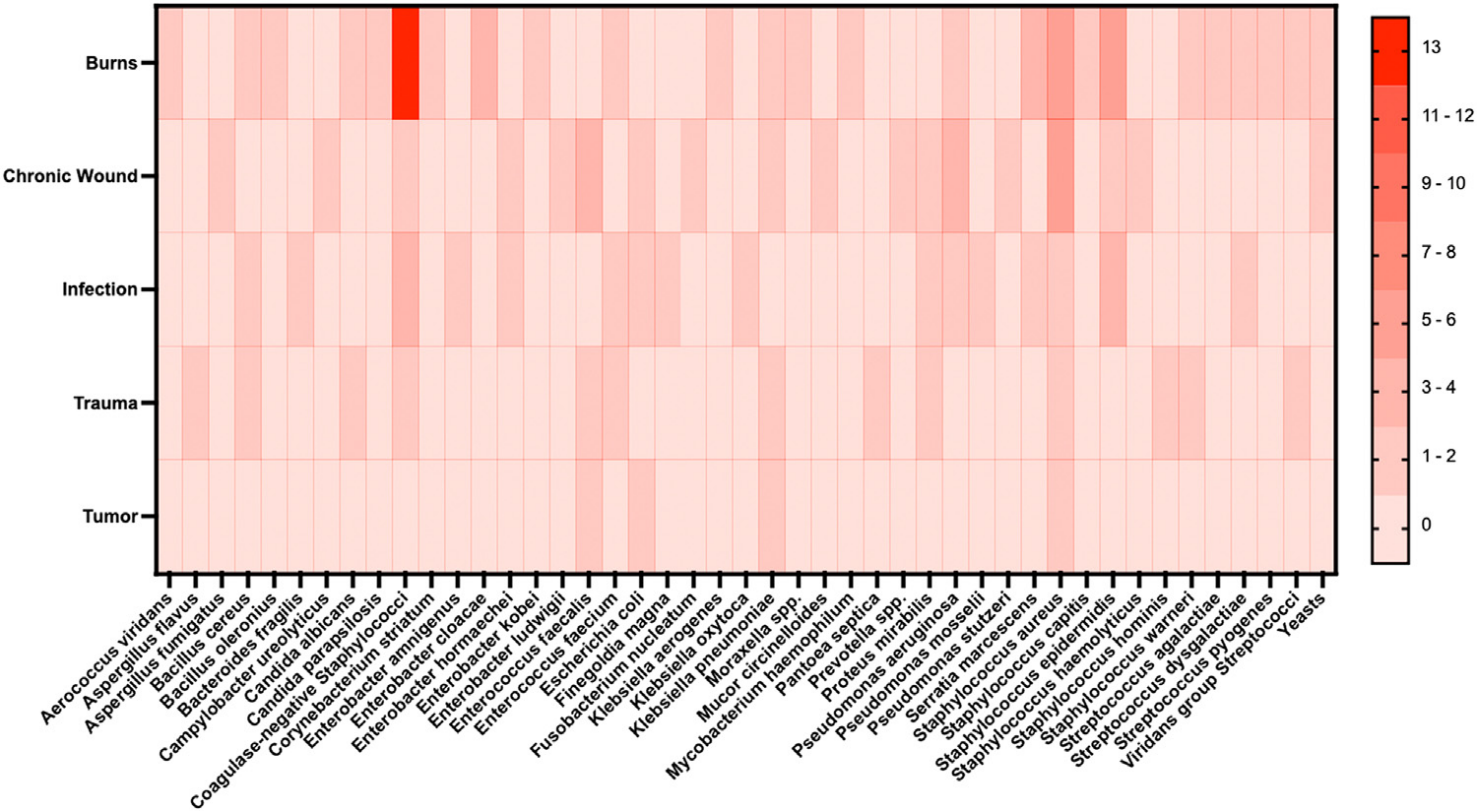
Distribution of positive wound swab cultures taken prior to BTM application according to wound etiology. Rows represent wound etiologies and columns represent individual microbial species. Color intensity corresponds to the frequency of isolation within each etiological subgroup, illustrating differences in microbial colonization patterns among burns, chronic wounds, infections, trauma, and tumor-related defects.

Microbiological findings are summarized in Fig. 4. Culture data prior to BTM application were available for all patients. Before BTM placement, 70.9% of wounds demonstrated contamination. The distribution of positive wound swab cultures taken prior to BTM application according to wound etiology is summarized in Fig. 5. Burns accounted for the greatest share of cases, comprising 42% of the cohort, followed by persistent chronic wounds (23%), infections (19%), trauma (12%) and tumors (4%).

Treatment results are reported in Table 3. Among all BTM covered wounds, a total BTM take (>90%) was noted in 77 cases (74.1%). A partial BTM take could be observed in 10 wounds (9.6%) with 17 wounds displaying BTM loss (16.3%). Postoperative hematoma was observed in 4 cases (3.8%) and infectious BTM complications at any postoperative interval were noted in 16 wounds (15.4%). On average, skin grafting occurred 25 days following BTM application (± 8.1). Overall, graft failure was observed in 6.7% (n = 7) while 77 wounds (74.1%) displayed satisfactory skin graft take >90%.

**Table 3:** Treatment results in all 104 wounds.

Variable	Total (n = 104)
BTM take rate, n (%)	
Total take (>90%), n (%)	77 (74.1)
Partial take, n (%)	9.(8.6)
None, n (%)	17 (16.3)
BTM infection, n (%)	16 (15.4)
BTM hematoma, n (%)	4 (3.8)
Skin graft take rate following total/partial BTM take, n (%)	
Total take (>90%), n (%)	62 (71.3)
Partial take, n (%)	19 (21.8)
None, n (%)	6 (6.9)
Time from BTM to skin grafting (days), (mean ± SD)	24.9 ± 8.1

BTM: Biodegradable Temporising Matrix; SD: standard deviation;.

In the comparative analysis between the BTM loss group (n = 17) and the BTM take group (n = 86), most baseline characteristics and comorbidities were similar between the two cohorts. There were no significant differences in age (49.7 ± 17.5 vs. 49.5 ± 18.0 years, p = 0.47), sex distribution (47.1%vs. 70.9% male, p = 0.06), arterial hypertension (47.1%vs. 34.9%, p = 0.34), renal insufficiency (23.5%vs. 29.1%, p = 0.64), diabetes mellitus (11.8%vs. 17.4%, p = 0.56), or active smoking (23.5%vs. 22.1%, p = 0.89).

Peripheral arterial disease was significantly more frequent in patients with BTM loss compared with those with successful BTM take (23.5%vs. 5.8%, p = 0.02). Similarly, exposed tendons were observed significantly more often in the BTM loss group (58.8%vs. 30.2%, p = 0.02). Other wound characteristics, including exposed bone (11.8%vs. 15.1%, p = 0.72), wound etiology, timing of BTM application, use of NPWT, and positive wound swabs prior to BTM application, did not differ significantly between groups. Overall, peripheral arterial disease and the presence of exposed tendons were the only variables significantly associated with BTM loss in this cohort (Table 4). Multivariable logistic regression however revealed that exposed tendons and peripheral arterial disease were not independently associated with BTM loss (Table 5). The multivariate model was statistically significant (p < 0.001).

**Table 4:** Comparative analysis of variables in BTM loss and BTM take cohorts.

Variable	BTM loss (*n* = 17)	BTM take (Partial & Total) (*n* = 86)	*p*-value
Age (years), (mean ± SD)	49.7 (±17.5)	49.5 (±18.0)	0.47
Male Gender, n (%)	8 (47.1)	61 (70.9)	0.06
Arterial hypertension, n (%)	8 (47.1)	30 (34.9)	0.34
Renal insufficiency (GFR < 90 ml/min), n (%)	4 (23.5)	25 (29.1)	0.64
Diabetes mellitus, n (%)	2 (11.8)	15 (17.4)	0.56
Active smokers, n (%)	4 (23.5)	19 (22.1)	0.89
Peripheral arterial disease, n (%)	4 (23.5)	5 (5.8)	**0.02**
Wound Etiology: Burns, n (%)	11 (64.7)	40 (46.5)	0.17
Wound Etiology: Chronic wounds, n (%)	4 (23.5)	11 (12.8)	0.25
Wound Etiology: Infection, n (%)	0 (0)	14 (16.3)	0.07
Wound Etiology: Trauma, n (%)	0 (0)	13 (15.1)	0.09
Wound Etiology: Tumor, n (%)	1 (5.9)	8 (9.3)	0.65
Exposed tendons, n (%)	10 (58.8)	26 (30.2)	**0.02**
Exposed bone, n (%)	2 (11.8)	13 (15.1)	0.72
BTM applied as primary wound coverage, n (%)	6 (35.3)	36 (41.9)	0.61
Wound > 2 weeks old at BTM application, n (%)	7 (41.2)	46 (53.5)	0.35
NWPT application, n (%)	8 (47.1)	48 (55.8)	0.51
Positive wound swab prior to BTM application, n (%)	14 (82.4)	58 (67.4)	0.22

GFR: glomerular filtration rate; BTM: Biodegradable Temporising Matrix; SD: standard deviation;.

**Table 5:** Multivariate logistic regression analysis of the risk factors for BTM loss.

Variable	OR	95% CI	p-value
Peripheral arterial disease	3.5	0.75 – 15.65	0.11
Exposed tendons	2.4	0.82 – 7.19	0.11

OR: odds ratio; CI: confidence interval;.

## Discussion

Split-thickness skin grafting alone, although widely employed, is limited by its inability to restore dermal volume in various wounds, often resulting in contracture, reduced pliability, and hypertrophic scarring, particularly over joints or in weight-bearing areas.^18^^,^^19^ By contrast, dermal regeneration templates aim to establish a vascularized neodermis prior to epidermal resurfacing, thereby improving biomechanical and aesthetic outcomes.

Initially developed for the management of burn defects,^20^ BTM has progressively been utilized in a wider spectrum of complex wounds. Reported indications now include traumatic injuries, soft tissue infections, chronic wounds and defects following oncologic resections.^21^, ^22^, ^23^ In this study, we describe our institutional experience using BTM for reconstruction in 104 wounds in 86 patients presenting with full-thickness skin defects of various etiologies.

Successful epithelial coverage was achieved in the majority of cases, with total BTM take exceeding 90% in 74.1% of wounds and satisfactory split-thickness skin graft take in 71.3%. Notably, only 16.3% of wounds demonstrated complete matrix loss, and overall skin graft loss following BTM integration was limited to 6.9%. These results are particularly meaningful given the complexity of the treated defects, with over half of patients presenting with deep structural exposure and nearly 71% of wounds demonstrating preoperative microbiological contamination.

The observed integration interval of approximately 25 days until grafting aligns with the expected biological timeline of neovascularization and dermal analog formation described for BTM.^12^^,^^15^ Clinically, the matrix demonstrated predictable incorporation, recognizable by uniform capillary refill and the characteristic dark pink appearance, permitting reliable transition to the second reconstructive stage.

One of the most clinically relevant findings is the comparatively low rate of infectious matrix-related complications (15.4%) despite a high prevalence of contaminated wounds at baseline (70.9%). The most frequently isolated microorganisms were coagulase-negative staphylococci (27.8%), *Staphylococcus aureus* (22.9%), *Staphylococcus epidermidis* (14.8%), *Pseudomonas aeruginosa* (11.5%), and *Escherichia coli* (9.8%). These organisms are commonly encountered in chronic wounds, burns, and traumatic soft tissue defects. Despite the high prevalence of preoperative wound colonization, neither positive wound swabs in general nor any specific microbial group demonstrated a significant association with BTM loss in our cohort. Due to the relatively limited number of BTM failures and infectious complications, statistical analysis of individual bacterial species as predictors of BTM infection or matrix loss was not sufficiently powered. Nevertheless, the successful integration observed in the majority of contaminated wounds supports the clinical applicability of BTM even in the presence of substantial microbial burden, provided that adequate debridement, wound bed preparation, and postoperative surveillance are ensured.

The synthetic polyurethane composition of BTM distinguishes it from xenogeneic or collagen-based dermal substitutes, which may theoretically be more susceptible to enzymatic degradation or biofilm-mediated destruction in contaminated environments.^23^^,^^24^ The absence of biologic components may contribute to its relative resilience in colonized wound beds. Previous studies have shown, that BTM appears to be associated with a longer integration and healing period than bovine-derived ADM Integra^Ⓡ^, while achieving higher graft take rates. Although infection rates were reported to be higher with BTM, no differences were observed in template take, reoperation requirements, or overall wound closure rates. Importantly, despite its prolonged healing timeline, BTM has shown advantages in terms of graft retention and reconstructive salvageability in the setting of infection compared with Integra^Ⓡ^, which may be attributable to the absence of biological components.^25^

Further, infection did not universally mandate complete matrix removal. In selected cases, localized management with irrigation, antiseptic therapy, and systemic antibiotics permitted salvage of the construct, allowing for a BMT salvage rate of 43.8% following BTM infection. This suggests that BTM may offer a degree of tolerance to bacterial burden when combined with meticulous wound bed preparation and vigilant postoperative monitoring. Given that burns constituted the largest etiological subgroup and are intrinsically associated with microbial colonization, these findings reinforce the applicability of BTM in acute burn reconstruction.

Univariate analysis identified exposed tendon and pre-existing peripheral arterial disease as factors significantly associated with BTM loss. The presence of exposed tendon likely reflects both local biological and mechanical challenges that compromise matrix integration. Tendons are relatively avascular structures with limited intrinsic capacity to support neovascularization, particularly when the paratenon is absent, thereby reducing the availability of a well-perfused wound bed necessary for scaffold incorporation.^26^ In addition, the anatomical locations in which tendon exposure commonly occurs, such as the distal extremities, are often times subject to increased shear forces and motion, which may further impair stable apposition of the matrix during the critical early phase of tissue ingrowth. In clinical practice, we try to minimize these detrimental effects by applying NPWT postoperatively. Additionally, we usually apply splints to immobilize the affected extremity to prevent movement that could further disrupt wound healing, to protect the wound edges from tension and separation, and to minimize the risk of further tissue damage or contamination. The splints are typically positioned to hold the joint in a neutral or functional position.

Similarly, peripheral arterial disease represents a systemic impairment of macro- and microvascular perfusion, directly limiting oxygen delivery, inflammatory cell recruitment, and angiogenic response within the wound bed.^27^ Given that successful BTM integration depends on timely and robust neovascularization of the polyurethane scaffold, compromised limb perfusion plausibly predisposes to partial or complete matrix failure. These findings underscore the importance of meticulous patient selection, aggressive optimization of limb perfusion where feasible, and heightened postoperative surveillance in individuals presenting with tendon exposure or underlying vascular insufficiency.

Nonetheless, these associations did not remain significant following multivariate adjustment. Neither exposed tendons (OR=2.41, 95% CI: 0.82–7.12, P = 0.11) nor peripheral artery disease (OR=3.5, 95% CI: 0.75–15.65, P = 0.11) reached statistical significance. However, the observed odds ratios suggest a clinically meaningful trend: patients with exposed tendons had approximately 2.4-fold and patients with peripheral artery disease approximately 3.5-fold increased odds of non-healing, indicating that these comorbidities may represent relevant risk factors in a challenging patient population. In particular, tendon exposure and peripheral arterial disease frequently coexist with other factors known to impair wound healing, such as large defect size, chronicity of the wound, or systemic vascular compromise. When these variables are considered simultaneously, the individual contribution of tendon exposure and peripheral arterial disease to BTM loss appears less pronounced. Consequently, while these factors may still indicate a higher-risk clinical context, they should not necessarily be interpreted as independent contraindications to the use of BTM, provided that meticulous wound bed preparation and careful postoperative monitoring are ensured.

The loss of statistical significance observed for tendon exposure and peripheral arterial disease after adjustment for multiple covariates likely reflects the complex interplay between these factors and other indicators of wound severity. Although both variables were associated with BTM failure in unadjusted analyses, they commonly occur alongside characteristics such as extensive tissue defects, anatomically distal lower-limb wounds, prolonged wound duration, and compromised local perfusion. As a result, their individual effects may be partly captured by these correlated variables, diminishing their apparent independent influence within the multivariable framework. In addition, the relatively small number of failure events in this cohort may have limited the ability of the analysis to identify independent predictors with sufficient statistical confidence.

From a practical standpoint, the findings indicate that tendon exposure and peripheral arterial disease should not necessarily preclude the use of BTM. Rather, they may serve as indicators of a more challenging wound milieu that warrants enhanced perioperative management. For individuals with peripheral arterial disease, evaluation of vascular status and optimization of blood flow should be undertaken whenever possible before reconstruction. In wounds involving exposed tendinous structures, strategies such as secure matrix stabilization, temporary immobilization, and the use of adjunctive negative-pressure wound therapy may help facilitate successful integration by reducing mechanical stress during the initial incorporation phase. Careful postoperative surveillance is advisable in both settings, as early recognition and treatment of emerging complications may improve the likelihood of successful matrix incorporation.

Still, our results compare favorably with previously published series evaluating BTM in burns, trauma, and chronic wounds, which report integration rates generally between 70 and 90% and infection rates ranging from 10 to 20%.^28^, ^29^, ^30^ The current cohort, characterized by substantial comorbidity including diabetes, renal insufficiency, arterial hypertension, and active smoking reflects a real-world population in whom reconstructive success is inherently more challenging.

Approximately 41% of the cohort consisted of patients with burn injuries, with a mean TBSA involvement of 31%. These patients frequently develop complications such as burn shock and sepsis, both of which can result in impaired tissue perfusion. In particular, reduced microcirculatory flow in the extremities may significantly compromise wound healing and graft integration. In this context, potential therapeutic strategies aimed at improving microcirculation may be of interest. For instance, agents such as pentoxifylline, already in use in our setting, could be considered as adjunctive approaches to enhance peripheral perfusion in these patients.

NPWT was applied in over 60% of cases following BTM placement. NPWT likely contributed to improved matrix adherence, exudate control, and minimization of shear forces, which is known to influence scaffold integration. Future multivariate analyses may clarify whether NPWT independently predicts improved BTM take, particularly in anatomically challenging regions such as the lower extremities, which represented the most frequently treated site in our cohort.

Nearly half of all wounds were older than two weeks at the time of BTM application, and a substantial proportion of patients exhibited systemic comorbidities known to impair wound healing. Despite these unfavorable baseline conditions, matrix integration was achieved in most cases. This suggests that once a macroscopically clean and stable wound bed is established, BTM can perform reliably even in patients with significant comorbidities.

Future prospective, controlled studies are needed to directly compare BTM with other available dermal substitutes in matched cohorts. Furthermore, long-term follow-up evaluating scar biomechanics, contracture rates, and patient-reported outcomes will be essential to determine whether early reconstructive advantages translate into sustained functional benefit.

## Limitations

Several limitations must be acknowledged. First, the retrospective design inherently limits causal inference and introduces potential selection bias. Second, the absence of a control group precludes direct comparison with alternative dermal substitutes or immediate autografting strategies. Further, etiology-specific and anatomical subgroup analyses present an important objective for future multicenter investigations. Third, long-term functional and aesthetic outcomes including scar quality, contracture rates, and patient-reported outcome measures were not systematically captured, limiting assessment of the true reconstructive superiority of staged dermal regeneration. We recommend that future prospective studies incorporate these standardized long-term outcome instruments. Additionally, economic considerations including cost-effectiveness relative to alternative reconstructive strategies were not evaluated and warrant future investigation.

## Conclusion

In this single-center cohort of complex full-thickness wounds, BTM demonstrated reliable integration, acceptable infection rates, and high rates of successful staged wound closure across diverse etiologies and high-risk patient profiles. Further, it has shown remarkable infection resilience despite pre-existing wound contamination. Only exposed tendons and pre-existing peripheral arterial disease were shown to be associated with BTM loss, although not as independent predictors.

## Funding

This research received no external funding.

## Institutional review board statement

The study was conducted according to the guidelines of the Declaration of Helsinki. It was approved by the Ethics Committee of Hannover Medical School (Ref.: 12,297-BO-K-2026).Manuscript type: Original Article.

## Declaration of competing interest

The authors declare no conflict of interest.
